# Oxygen Deprivation and the Cellular Response to Hypoxia in Adipocytes – Perspectives on White and Brown Adipose Tissues in Obesity

**DOI:** 10.3389/fendo.2015.00019

**Published:** 2015-02-19

**Authors:** Paul Trayhurn, Suliman Yousef Alomar

**Affiliations:** ^1^Clore Laboratory, Buckingham Institute for Translational Medicine, University of Buckingham, Buckingham, UK; ^2^College of Science, King Saud University, Riyadh, Saudi Arabia; ^3^Obesity Biology Unit, Institute of Ageing and Chronic Diseases, University of Liverpool, Liverpool, UK

**Keywords:** adipocyte, brite cell, brown adipose tissue, hypoxia, lactate, uncoupling protein-1, oxygen, white adipose tissue

## Abstract

Relative hypoxia has been shown to develop in white adipose tissue depots of different types of obese mouse (genetic, dietary), and this leads to substantial changes in white adipocyte function. These changes include increased production of inflammation-related adipokines (such as IL-6, leptin, Angptl4, and VEGF), an increase in glucose utilization and lactate production, and the induction of fibrosis and insulin resistance. Whether hypoxia also occurs in brown adipose tissue depots in obesity has been little considered. However, a recent study has reported low pO_2_ in brown fat of obese mice, this involving mitochondrial loss and dysfunction. We suggest that obesity-linked hypoxia may lead to similar alterations in brown adipocytes as in white fat cells – particularly changes in adipokine production, increased glucose uptake and lactate release, and insulin resistance. This would be expected to compromise thermogenic activity and the role of brown fat in glucose homeostasis and triglyceride clearance, underpinning the development of the metabolic syndrome. Hypoxia-induced augmentation of lactate production may also stimulate the “browning” of white fat depots through recruitment of UCP1 and the development of brite adipocytes.

## Introduction

Oxygen is rarely considered as an essential nutrient in mammals and this is primarily because it is delivered through the lungs rather than in the diet via the gastrointestinal tract. However, at a cellular level, it is a key nutritional factor without which oxidative metabolism – particularly oxidative phosphorylation in mitochondria – cannot take place. The delivery of O_2_ to cells from the circulation is via a specific carrier protein, hemoglobin, paralleling the general mechanism by which a number of nutrients are delivered. At a whole-body level, the consequences of, and adaptations to, low O_2_ tension have been extensively investigated. This is especially so in relation to high altitude and in response to the O_2_ deprivation experienced during deep-sea diving. The effect of a lack of O_2_ has also been widely considered in relation to disorders such as obstructive pulmonary disease where lung function is impaired, and during the periodic reduction in O_2_ delivery in sleep apnea (1).

These states impact on the provision of O_2_ to the body as a whole. Changes in O_2_ level in individual tissues, specifically a reduction, can occur even when the total supply is not compromised. Examples include at the site of wounds during healing from tissue damage, and in the center of solid tumors (2–4). There is growing recognition that low O_2_ tension, indeed overt hypoxia, can occur in tissues with important implications for cellular function. White adipose tissue (WAT) is one site where hypoxia has been demonstrated and this occurs with the expansion of adipocyte size and total adipose mass in obesity (5–8).

In this article, we provide a perspective on the effects of hypoxia on the function of white adipocytes and consider, in particular, whether hypoxia also occurs in brown adipose tissue (BAT). It has been argued, of course, that collectively white and brown fat constitute a single adipose organ (9). We speculate on the effects that reduced O_2_ tension might have on the function of brown adipocytes and the possible implications for obesity and the metabolic syndrome.

## White Adipose Tissue

White adipose tissue, which is a major component of total body composition comprising at least 40% of body weight in obese adults, is the principal site of fuel storage and has major additional functions including in particular that of being a key endocrine organ. White adipocytes, the signature cell of WAT, are major secretory cells, releasing a multiplicity of lipid and protein moieties additional to the fatty acids mobilized by lipolysis (8, 10). Several pleiotropic protein hormones are synthesized and secreted by white adipocytes, the most prominent being leptin and adiponectin (11, 12). These hormones were first discovered in adipose tissue, and the identification of leptin firmly established white adipocytes as endocrine cells.

Subsequent investigations demonstrated that white fat cells secrete a large and diverse range of “adipokines” (13–16), with proteomic studies suggesting that there are several hundred such protein factors (17). Multiple adipokines are associated with inflammation, including classical cytokines and chemokines such as TNFα, IL-1β, IL-6, and MCP-1 (13–15). The synthesis and release of a number of inflammation-related adipokines increase in obesity, adipose tissue depots becoming “inflamed” with the inflammatory state underpinning the development of obesity-associated diseases – particularly insulin resistance and the metabolic syndrome (10, 15, 18, 19).

Adipokines are not the only inflammation-related moieties produced by white adipocytes, various prostaglandins also being released (8). These lipid factors have received much less attention in considering the inflammatory response in adipose tissue. Indeed, there has been markedly less focus overall on the lipid secretions from the tissue – apart from fatty acids. This is despite white adipocytes releasing multiple lipid moieties, which include endocannabinoids (such as anandamide), cortisol (by the conversion of cortisone), vitamin D_3_, vitamin A, and cholesterol – some of which are sequestered within WAT rather than being synthesized *de novo* (1, 8).

## Hypoxia in White Adipose Tissue

Hypoxia is one of the central mechanisms postulated to explain the development of inflammation and the subsequent metabolic dysfunction of WAT in obesity (10, 20, 21). This is linked to the growing recognition that O_2_ levels are far from the same in all tissues, and neither are they constant. For example, while the general level of tissue oxygenation (pO_2_) is 45–50 mmHg, that of the thymus is 10 mmHg, while for the brain it is as low as 0.4–8 mmHg (3, 22) – and the center of solid tumors can be essentially anoxic (2, 3). In the case of WAT, it was proposed that as fat mass expands in the obese, large adipocytes become distant from the vasculature and areas of O_2_ deprivation occur (10, 20). Hypoxia was subsequently demonstrated in WAT in various obese rodents – genetically obese *ob/ob* and *KKAy* mice, and mice with diet-induced obesity (5–7). Two distinct experimental approaches documented hypoxia in these obese mice; in one, which utilized the hypoxia marker pimonidazole, low pO_2_ was shown qualitatively. Quantitative measurements have been obtained using needle-type O_2_ sensors, and these found between 2- and 3.5-fold reductions in pO_2_ in WAT of *ob/ob* and dietary obese mice relative to lean controls – down to 15 mmHg compared with 45–50 mmHg, for obese and lean animals, respectively (5–7, 23).

In contrast to the clear evidence for hypoxia in WAT in rodent obesity, the situation in obese humans is more problematic. Several earlier studies demonstrated reduced pO_2_ in human obesity, consistent with the more limited vascularization in the obese – limited since the blood supply to WAT does not rise despite the substantial increase in the size of the fat depots (24–26). Proportionally, the vascular supply is reduced per unit adipose mass in obese humans, capillary density being lower than in the lean. A further key observation is that obese humans do not exhibit the post-prandial rise in blood flow to WAT that occurs in lean individuals (27, 28). While the degree of hypoxia is the modest in the human studies recording reduced O_2_ tension, two recent reports have found no evidence for lower O_2_ levels (28, 29). Indeed, in one study, hyperoxia rather than hypoxia was noted (28). In the study reporting neither hypoxia nor hyperoxia, reduced delivery of blood and lowered consumption of O_2_ were observed, nevertheless; in addition, there was a net release of lactate, consistent with anaerobic glycolysis (29). At present, it is not evident why such divergent results have been obtained, although methodological issues may be important.

In parallel with *in vivo* investigations on the O_2_ tension of WAT in obesity, extensive *in vitro* studies on the molecular and cellular response to hypoxia of adipocytes (human and rodent) in culture have been undertaken (5, 6, 8, 30–32). These initially focused on the expression (and in some cases release) of key adipokines, both those directly linked to inflammation and the signature adipocyte hormones leptin and adiponectin. The expression and release of leptin, VEGF, serum amyloid A, Angptl4, and IL-6, for example, are increased in the presence of hypoxia, while adiponectin production is reduced (5, 6, 8, 30–32). This is indicative of the induction of an inflammatory state. Microarray studies have shown that the expression of up to 1,300 genes is hypoxia-sensitive (~50% upregulated/~50% downregulated) in human adipocytes, indicating an extensive effect of low O_2_ tension on gene expression in these cells (33, 34). The genes encoding proteins in key pathways and processes modulated by low pO_2_ include those associated with glycolysis, mitochondrial (oxidative) metabolism, cell death, and inflammation (33, 34). Some of these changes clearly reflect a switch from aerobic to anaerobic metabolism.

Alterations in gene expression in hypoxia, not all of which are necessarily primary, are, of course, significant, but it is changes in metabolic function that are of greatest importance. Studies with 2-deoxy-d-glucose demonstrate that glucose uptake into adipocytes is stimulated by hypoxia, and this is a transport-mediated process (35, 36). The key transporter involved is GLUT1, responsible for basal glucose uptake in many cells, since hypoxia induces a substantial increase in its expression and amount (35, 37). There is a parallel elevation in the release of lactate from hypoxic adipocytes, this also involving the recruitment of a specific transport protein – MCT1, a monocarboxylate transporter (38). Increases in glucose uptake and lactate release are indicative of the stimulation of anaerobic glycolysis and the levels of glycolytic enzymes, as well as the expression of genes linked to glycolysis, are raised in hypoxic adipocytes (33, 34, 39).

Other functional changes in fat cells in response to low pO_2_ include the rates of lipolysis and lipogenesis. Lipolysis is reported as both increased and unchanged; however, the evidence for the former appears stronger (23, 30, 40). Lipogenesis is reduced in hypoxia, as is the uptake of fatty acids, and the effect of reduced pO_2_ on these processes may be mediated through an inhibition of hexosamine biosynthesis (23, 40). A major direct effect of hypoxia on adipocyte function is the induction of insulin resistance. Thus, there is a loss of insulin-stimulated glucose uptake, as indicated by 2-deoxy-d-glucose studies, and an inhibition of the insulin signaling pathway – including a lack of phosphorylation of insulin receptor-β and insulin receptor substrate (23, 36). Insulin resistance may well be the key element in adipose tissue dysfunction induced by hypoxia in obesity.

A further area of adipocyte function in which low O_2_ tension plays a role is in the induction of a pro-fibrotic transcriptional program, fibrosis resulting from the disruption of the extracellular matrix (41, 42). Fibrosis is now considered as part of adipose tissue dysfunction in obesity, and the expression of a number of genes encoding proteins of the extracellular matrix, including specific collagens, is altered in adipocytes by hypoxia (42).

Other cells in WAT additional to adipocytes are sensitive to hypoxia, including macrophages and pre-adipocytes. In the case of macrophages, the inflammatory state is stimulated by low pO_2_ (6, 43, 44). A major effect of hypoxia on pre-adipocytes is to inhibit the differentiation of these precursor cells into adipocytes, this involving a downregulation of PPARγ through the key hypoxia-inducible transcription factor, HIF-1 (8). A particularly intriguing effect of low pO_2_ on pre-adipocytes is their conversion into leptin-secreting, endocrine cells; prior to their differentiation into mature adipocytes, pre-adipocytes do not express the *Lep* gene (45). This effect is evident with overt hypoxia (1% O_2_) and an important question is at what O_2_ level pre-adipocytes express the *Lep* gene and secrete functional leptin. It has been argued that the lack of leptin production by pre-adipocytes cultured under normal conditions may be an artifact, reflecting an inhibitory effect of hyperoxia since the cells are customarily incubated under 21% O_2_ (1).

This raises the broader question of the appropriate O_2_ tension at which to culture or incubate cells in general. Again, the 21% O_2_ present in air is usually employed, but this represents a considerably higher O_2_ tension than the 45–50 mmHg (~7% O_2_ equivalent) that most tissues normally experience. A study on human adipocytes exposed to different levels of O_2_ indicates that at physiological levels of oxygenation the cells exhibit increased expression of those adipokine genes that are hypoxia-sensitive and there is elevated secretion of the functional protein relative to 21% O_2_ (37). Similarly, glucose uptake and lactate release, together with the expression of their respective transporters, are greater at 7% O_2_ than at so-called “normoxia” (37). Thus, at physiological levels of O_2_ adipocytes (and other cells) are relatively hypoxic and exhibit some of the cellular changes characteristic of this state – though greater effects are evident with 1% O_2._

## Brown Adipose Tissue

There has been little consideration of whether hypoxia occurs in BAT in obesity, and if so whether the key functions of brown adipocytes are modified. The primary role of BAT as a specialized organ for generating heat by non-shivering mechanisms (46, 47) is dependent on the extensive vascularization that is evident in the tissue. A substantial blood supply is required to provide O_2_ and other nutrients to fuel thermogenesis and to rapidly distribute heat throughout the body – in part, to ensure that BAT is not itself damaged through an inappropriate elevation in local temperature. Heat constitutes, of course, the primary form of cross-talk from BAT to other tissues.

Heat is generated in brown adipocytes through the uncoupling of oxidative phosphorylation such that the proton gradient across the inner mitochondrial membrane is dissipated as heat rather than being coupled to ATP synthesis (47, 48). This proton conductance pathway is dependent on, and regulated by, the 32,000 mol. wt. mitochondrial uncoupling protein, UCP1 (49, 50). Thermogenesis is stimulated by noradrenaline released from the sympathetic nervous system, with the sympathetic system also playing a central role in the development of thermogenic capacity (51). By virtue of its role in heat production for thermoregulation, BAT is prominent in small rodents adapted to the cold, in the newborn of precocial species (such as lambs and cattle), and in hibernators (such as ground squirrels). BAT is also a site of diet-induced thermogenesis with this form of adaptive heat production being a component in the regulation of energy balance and body fat (52–54). Throughout the 1980s, BAT was a key focus of obesity research, reflecting the proposition that reduced thermogenesis is important in the etiology of the obese state (54–56).

The recent renewed interest in BAT has followed from reports in which definitive evidence for metabolically active brown fat in adult humans has been presented. The application of fluorodeoxyglucose positron emission tomography (FDG-PET) has identified sites of fat tissue that exhibit high glucose uptake (57), and together with immunostaining for UCP1 has unambiguously identified BAT in adult humans (58–60). Subsequent reports have demonstrated that the abundance and thermogenic activity of brown fat in humans exhibits considerable plasticity, FDG-PET investigations indicating that acute exposure to cold activates BAT in humans (59, 61–63). Importantly, in relation to energy balance, observations from FDG-PET indicate an inverse relationship of BAT activity to age and BMI, the tissue being less active, or less evident, in older subjects and in the obese (64–66).

While the central role of BAT is to generate heat, brown adipocytes produce and secrete specific protein signals – in effect adipokines similar to white fat – and these include leptin and IL-6 (67–69). The actions of these adipokines is likely to be essentially autocrine/paracrine, rather than endocrine, since the contribution from brown fat to circulating levels will be small relative to that of WAT. Whether the “adipokinome” in brown adipocytes is extensive is unknown. BAT is increasingly recognized to have direct metabolic functions, FDG-PET studies showing that the tissue has a high rate of glucose uptake, which can be stimulated by insulin as well as cold exposure (59, 61–63). Recent reports have demonstrated that brown fat is important in glucose homeostasis with the tissue being proposed as a major organ in glucose disposal and insulin sensitivity (70, 71). A further metabolic role now attributed to the tissue is in triglyceride clearance, particularly during cold exposure (70, 72).

An apparent substantive contribution of BAT to glucose homeostasis, insulin sensitivity and lipid clearance has led to the suggestion that reduced activity of the tissue may be implicated in the development of the metabolic syndrome, with re-activation potentially leading to a reversal of the syndrome (72, 73).

## Hypoxia in Brown Adipose Tissue

As noted above, BAT is highly vascularized consistent with the need to supply O_2_ and other nutrients to meet the metabolic demands of thermogenesis. Studies on regional blood flow in the late 1970s indicated that the tissue is responsible for >50% of total non-shivering thermogenesis in rats acclimated to the cold, with up to one-third of the total cardiac output being directed toward BAT during maximal thermogenesis (74, 75). In the case of Sultzer’s vein, which drains the main interscapular depot in rodents, O_2_ extraction appears almost total (76).

Increased lipid deposition occurs in brown adipocytes in obesity with BAT enlarging in obese animals, similar to the expansion of white fat depots. The increase in adipocyte size in BAT raises the possibility that pockets of reduced O_2_ tension develop in the tissue in the obese and that this leads to the inhibition of the acute thermogenic response. Indeed, hypoxia may have major effects on gene expression and cellular metabolism in brown adipocytes, as in white, BAT function being compromised through the development of insulin resistance and reduced thermogenesis. BAT thermogenesis is recognized to be reduced in obese rodents, as indicated by measurements of regional blood flow and O_2_ utilization in response to noradrenaline, as well as from studies on the amount and activity of UCP1 (55, 56, 77).

Hypoxia has been observed through pimonidazole staining in BAT of lean mice exposed to the cold (4°C), but it is not evident in mice acclimated to the warm (30°C); *Ucp1* knockout indicates that it occurs only with thermogenic activity (78). A recent study has directly examined whether there is hypoxia in brown fat in the obese and described evidence for vascular “rarefaction” in BAT of mice with diet-induced obesity, leading to a “whitening” of the tissue with enlarged lipid droplets together with mitochondrial dysfunction and loss (79). Hypoxia was demonstrated in the obese by pimonidazole staining and with a fiber-optic O_2_ sensor, the latter indicating a >5-fold reduction in pO_2_ compared with BAT of lean mice – a greater degree of hypoxia than occurred in WAT of the same animals. Among the other changes observed in the obese BAT were increases in the level of the hypoxia-sensitive transcription factor subunit, HIF-1α, and falls in *Ucp1* expression and β-adrenergic signaling (79).

**Figure 1:**
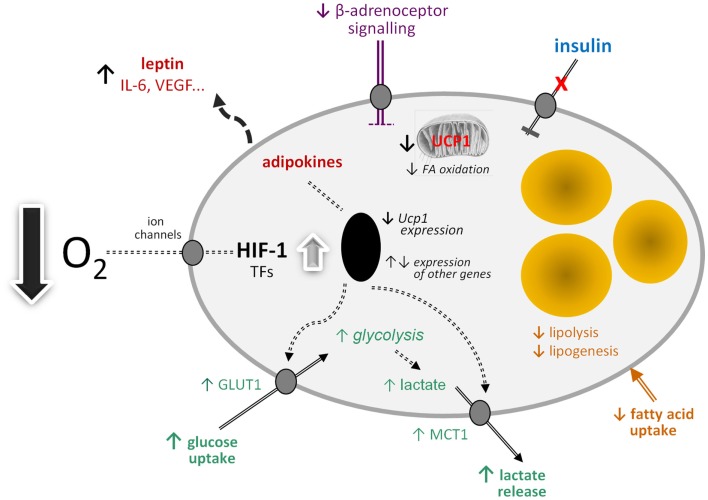
**Schematic view of the potential effects of hypoxia on brown adipocyte function**. FA, fatty acid; GLUT1, facilitative glucose transporter-1; HIF-1, hypoxia-inducible factor-1; IL-6, interleukin-6; MCT1, monocarboxylate transporter-1; TFs, transcription factors; *Ucp1*, uncoupling protein-1 (gene); VEGF, vascular endothelial growth factor.

By analogy with white adipocytes, there are a number of other potential effects of hypoxia on brown adipocytes in obesity (Figure 1). These include changes in the transcription of a range of hypoxia-sensitive genes, including those encoding adipokines; the expression of leptin, VEGF, and IL-6, for example, may increase. Glucose uptake would be expected to rise through increased expression and recruitment of GLUT1. Insulin-stimulated glucose uptake, on the other hand, may fall through the development of insulin resistance. A loss of insulin sensitivity is evident in BAT of obese animals and occurs in *ob/ob* mice soon after weaning (80, 81). This development of resistance is associated with a reduction in acute thermogenesis, and its reversal with ciglitazone restores the thermogenic response of BAT to cold (82).

Further potential metabolic changes in brown adipocytes in response to low O_2_ tension include the induction of fibrosis and a fall in lipogenesis and fatty acid uptake. Reduced lipid uptake as well as the induction of insulin resistance would be consistent with hypoxia being a mechanism for the development of the metabolic syndrome in obesity, given the postulated role of BAT in glucose homeostasis and triglyceride clearance. This, together with direct effects on limiting thermogenesis itself, suggests that hypoxia has the potential to severely compromise BAT function with important implication for metabolic homeostasis and disease processes.

If mitochondrial loss and dysfunction are induced in BAT by hypoxia and glucose utilization is elevated, this would indicate a switch to anaerobic glycolysis. One of the consequences of such a switch would be an increase in lactate production and release, involving the further recruitment of the MCT1 transporter. Lactate has recently emerged as an important metabolic signal, the functions attributed to it including the stimulation of inflammation in macrophages and – importantly – mediation of the anti-lipolytic effect of insulin (83, 84). This anti-lipolytic action is via the GPR81 receptor, which is expressed principally in adipose tissues (84).

## Hypoxia and the Recruitment of Brite Adipocytes

A further major function recently identified for lactate is in the “browning” of white adipocytes (85). The concept of browning has emerged with the recognition that there is a third type of adipocyte in addition to white and brown fat cells – the brite (or beige) adipocyte. Brite cells are found especially in white fat depots and have many, though not all, of the molecular characteristics of brown adipocytes (86, 87). The key feature shared by brite and brown adipocytes is the presence of UCP1, providing the potential for thermogenesis (86, 88).

Cold exposure of mice results in an elevation in circulating lactate levels and *Mct1* gene expression is stimulated in both BAT and WAT (85). Treatment with lactate, *in vivo* or *in vitro*, induces *Ucp1* gene expression in white adipocytes and the expression of genes associated with fatty acid oxidation and mitochondrial function, indicative of the recruitment of a thermogenic profile (85). Lactate does not lead to the induction of UCP1 in white fat cells through the GPR81 receptor, but rather by modulation of MCT activity; preventing lactate influx by pharmacological inhibition of MCTs suppressed the lactate-induced expression of *Ucp1*. Correspondingly, knockdown of MCT4, the transporter responsible for lactate efflux, strongly enhanced *Ucp1* expression in adipocytes. It is suggested that the lactate-induced expression of *Ucp1* in white adipocytes, which is independent of HIF-1α, is a mechanism for reducing oxidative stress resulting from high intracellular lactate levels (85).

**Figure 2:**
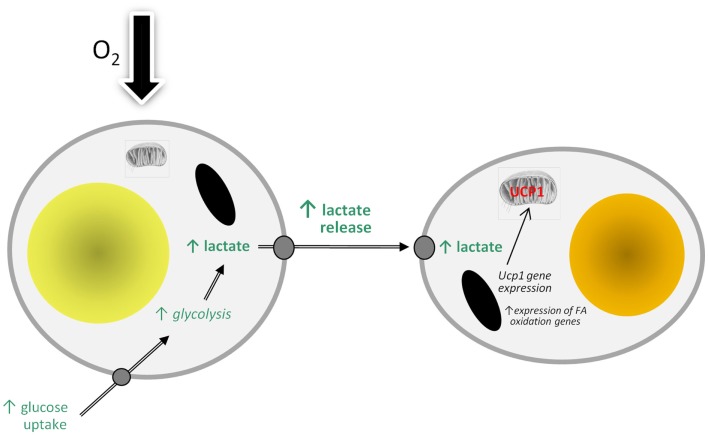
**Model of how hypoxia may lead to the recruitment of brite adipocytes and the “browning” of white adipose tissue depots through stimulating the production and release of lactate**. FA, fatty acid; UCP1, uncoupling protein-1.

There are, of course, an increasing number of agents which have been reported to stimulate “browning” (89), but lactate is of particular interest because of its direct physiological relevance. WAT is an important site of lactate production and the levels in the tissue are increased in obesity (5, 90). A model can be proposed in which lactate production rises in WAT in obesity in response to developing hypoxia as large adipocytes switch from aerobic to anaerobic metabolism (Figure 2). The lactate then stimulates *Ucp1* expression in neighboring white adipocytes, through an autocrine/paracrine action, leading to the recruitment of brite cells and the emergence of the “browning” phenotype in WAT. This could reflect a counter-regulatory response with the accumulation of large adipocytes resulting, via a hypoxia-induced elevation in lactate, in the production of thermogenic cells, which would oxidize lipid. However, the extent to which brite adipocytes may contribute to thermogenesis is debatable, with an extensive vascularization being one of the factors required for clusters of brite cells to make a significant contribution to adaptive heat production.

## Coda

The effects of hypoxia on white adipocytes are extensive, ranging from the stimulation of the production of adipokines linked to inflammation to the induction of insulin resistance. Hypoxia also occurs in BAT in obesity, compromising thermogenic activity and potentially the role of the tissue in metabolic homeostasis. Mitochondrial loss and dysfunction, insulin resistance, fibrosis, increased glucose uptake, and lactate production may all occur in BAT following hypoxia, paralleling many of the changes in WAT induced by low pO_2_. Hypoxia-induced increases in lactate production by white adipocytes in obese WAT provides a signal for the induction of browning such that *Ucp1* is expressed and brite fat cells recruited.

It is suggested that reduced O_2_ tension may be a critical influence on all forms of adipose tissue in obesity, profoundly altering the function of classical white and brown adipocytes and stimulating the development of brite fat cells.

## Conflict of Interest Statement

The authors declare that the research was conducted in the absence of any commercial or financial relationships that could be construed as a potential conflict of interest.
